# B‐Site Co‐Alloying with Germanium Improves the Efficiency and Stability of All‐Inorganic Tin‐Based Perovskite Nanocrystal Solar Cells

**DOI:** 10.1002/anie.202008724

**Published:** 2020-09-25

**Authors:** Maning Liu, Hannu Pasanen, Harri Ali‐Löytty, Arto Hiltunen, Kimmo Lahtonen, Syeda Qudsia, Jan‐Henrik Smått, Mika Valden, Nikolai V. Tkachenko, Paola Vivo

**Affiliations:** ^1^ Chemistry and Advanced Materials Group Faculty of Engineering and Natural Sciences Tampere University P.O. Box 692 33014 Tampere Finland; ^2^ Surface Science Group Faculty of Engineering and Natural Sciences Tampere University P.O. Box 692 33014 Tampere Finland; ^3^ Faculty of Engineering and Natural Sciences Tampere University P.O. Box 692 33014 Tampere Finland; ^4^ Laboratory of Molecular Science and Engineering Åbo Akademi University Porthansgatan 3–5 20500 Turku Finland

**Keywords:** lead-free, perovskite nanocrystals, solar cells, time-resolved photoluminescence, ultrafast transient absorption spectroscopy

## Abstract

Colloidal lead‐free perovskite nanocrystals have recently received extensive attention because of their facile synthesis, the outstanding size‐tunable optoelectronic properties, and less or no toxicity in their commercial applications. Tin (Sn) has so far led to the most efficient lead‐free solar cells, yet showing highly unstable characteristics in ambient conditions. Here, we propose the synthesis of all‐inorganic mixture Sn‐Ge perovskite nanocrystals, demonstrating the role of Ge^2+^ in stabilizing Sn^2+^ cation while enhancing the optical and photophysical properties. The partial replacement of Sn atoms by Ge atoms in the nanostructures effectively fills the high density of Sn vacancies, reducing the surface traps and leading to a longer excitonic lifetime and increased photoluminescence quantum yield. The resultant Sn‐Ge nanocrystals‐based devices show the highest efficiency of 4.9 %, enhanced by nearly 60 % compared to that of pure Sn nanocrystals‐based devices.

## Graphical abstract

CsSn_0.6_Ge_0.4_I_3_ nanocrystals have been synthesized for the first time by a B‐site co‐alloying strategy. The introduction of Ge effectively decreases the high density of intrinsic Sn defects, resulting in an extended excitonic lifetime and enhanced solar cell performance. The stability of the new nanocrystals also improves owing to the effective protection of Sn^2+^ against oxidation.

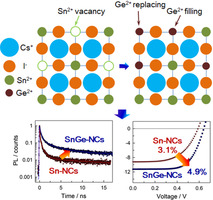

## Introduction

Colloidal halide perovskite nanocrystals (PNCs), with their size‐tunable narrow‐band emission, higher photoluminescence quantum yields (PLQY) than the corresponding bulk crystals, and enhanced surface‐to‐volume ratio,^[1]^ are promising candidates for the next‐generation optoelectronics including PVs,[^2^, ^3^] light‐emitting diodes,[^4^, ^5^] photodetectors,^[6]^ and lasers.^[7]^ Because of the intrinsic instability in ambient conditions of organic cations (for example, CH_3_NH_3_^+^) at the A site of a typical ABX_3_ perovskite structure,^[8]^ all‐inorganic lead (Pb) based PNCs, such as CsPbX_3_ (X=Cl, Br, or I), are preferred to pursue high stability while retaining the advanced optical properties, for example, the high PLQY.^[9]^ However, toxic Pb is still the key constituent of the most efficient halide perovskite materials, thus impeding PNCs widespread commercialization.

Tin (Sn) is one of the most promising alternative candidates to replace Pb, being its closest analogue with similar structural and electronic properties.[^10^, ^11^] As a result, the most efficient Pb‐free perovskite solar cells (PSCs) to date always include Sn in the perovskite composition.[^12^, ^13^, ^14^] However, integrating Sn in spatially confined nanocrystals (NCs) has been rarely successful because of its high instability. Sn^2+^ (the B site in the perovskite structure) is rapidly oxidized to Sn^4+^ in ambient conditions.[^15^, ^16^] Furthermore, the high density of surface vacancies^[17]^ result in the low photoluminescence quantum yield (PLQY) of Sn‐based PNCs. As another promising Pb‐free candidate, germanium (Ge) is a defect‐tolerant and nontoxic semiconductor still highlighted by the limited studies.[^18^, ^19^] Among the synthesized materials, CsGeI_3_ has a direct band gap (*E*_g_) of 1.6 eV, which is suitable for PVs. When compared to CsSnI_3_, CsGeI_3_ does display, however, inferior optoelectronic properties owing to the lone‐pair effect^[20]^ and relatively larger *E_g_*. Inspired by the heterovalent B‐site co‐alloying strategy for Pb and Sn, several reports have proposed to mix Sn with Ge for a new candidate of mixture lead‐free perovskite.[^21^, ^22^, ^23^, ^24^, ^25^, ^26^] By first‐principles computations, Ju et al. predicted several potentially promising mixed Sn‐Ge halide perovskites as light absorbers for solar cells.^[27]^ Ma et al. presented a comprehensive theoretical study on the mixed vacancy‐ordered inorganic double perovskite Cs_2_Sn_(1−*x*)_Ge_*x*_I_6_, indicating that the concentration of Ge doping severely influences the thermodynamic, electronic, and mechanical properties.^[25]^ Ito et al. experimentally showed an improved power conversion efficiency (PCE) of 4.48 % for FA_0.75_MA_0.25_Sn_1−*x*_Ge_*x*_I_3_‐based solar cells with a 5 % Ge doping into the perovskite, compared with that (3.31 %) of pure Sn‐based ones.^[28]^ Later on, Chen et al. reported the first synthesis of CsSn_0.5_Ge_0.5_I_3_ bulk crystals via a vapor method, showing a relatively high PCE of 7.11 % but employing a complex, non‐scalable, and expensive synthetic route.^[29]^ However, there are no examples of mixed Sn‐Ge perovskite nanocrystals.

Here, we pioneered the synthesis of all‐inorganic Ge alloyed CsSnI_3_ perovskite nanocrystals (Sn‐PNCs) via a facile and low‐cost solution process. The novel CsSn_0.6_Ge_0.4_I_3_ perovskite nanocrystals (SnGe‐PNCs) exhibit significantly improved optical and photophysical properties compared to those of reference Sn‐PNCs. The stability of as‐formed CsSn_0.6_Ge_0.4_I_3_ has also been effectively enhanced upon the introduction of Ge. The partial replacement of Sn atoms by Ge atoms in the nanostructure can effectively fill the high density of Sn vacancies and reduce the surface traps, resulting in a longer excitonic lifetime and improved PLQY. In this work, the PCE (4.9 %) of the PSCs employing CsSn_0.6_Ge_0.4_I_3_ nanocrystals is enhanced by nearly 60 % compared to that (3.1 %) of CsSnI_3_‐based devices.

## Results and Discussion

### Synthesis of CsSn_*x*_Ge_1−*x*_I_3_ NCs

**Figure 1:**
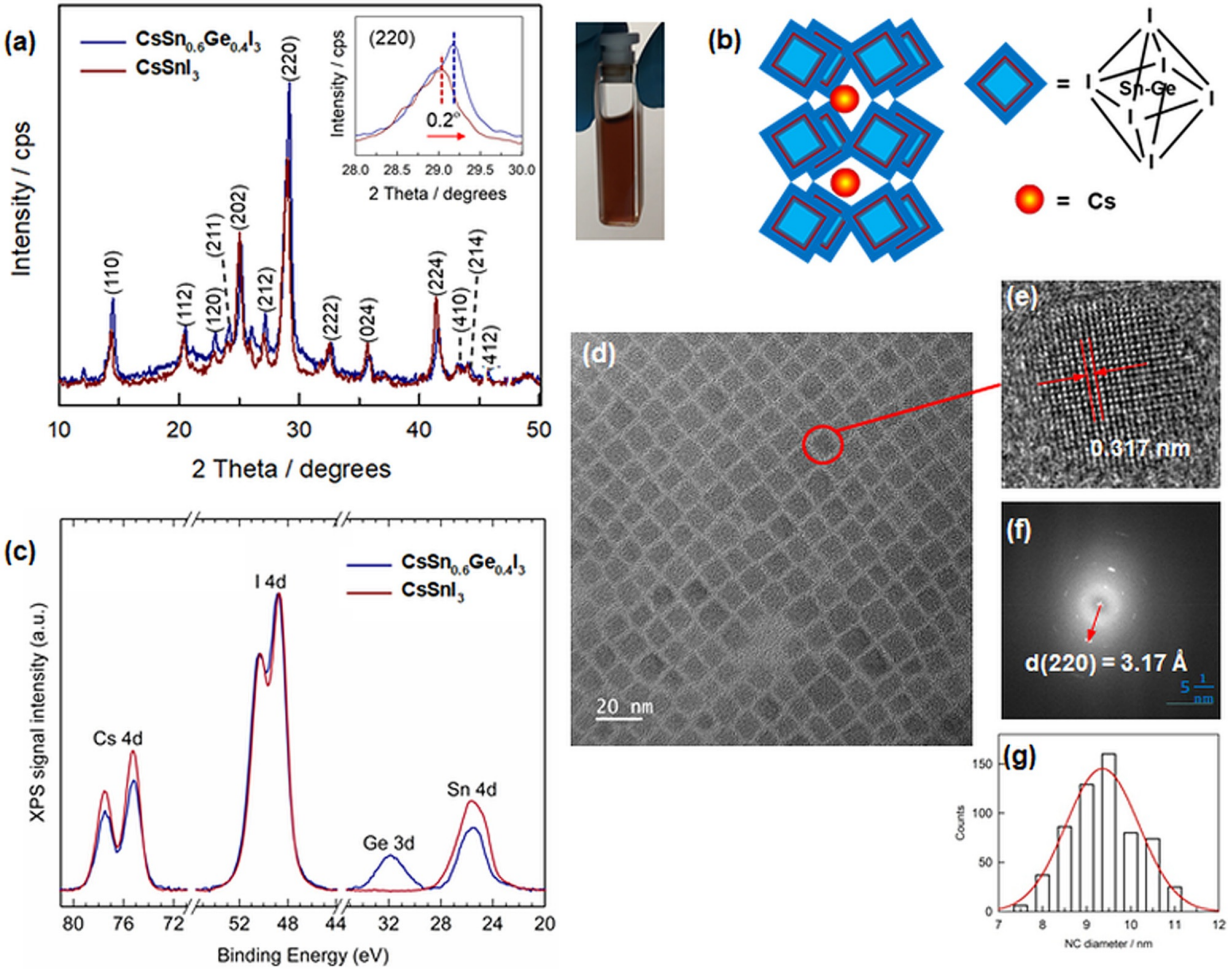
a) X‐ray diffraction patterns of the as‐prepared CsSn_0.6_Ge_0.4_I_3_ NCs and reference CsSnI_3_ NCs. The side picture shows the appearance of CsSn_0.6_Ge_0.4_I_3_ NCs in suspension. b) Illustration of a unit cell of CsSn_0.6_Ge_0.4_I_3_ NCs in orthorhombic crystal structure. c) X‐ray photoelectron spectroscopy (XPS) analysis of CsSnI_3_ NCs and CsSn_0.6_Ge_0.4_I_3_ NCs. d) Transmission electron microscopy (TEM) image of CsSn_0.6_Ge_0.4_I_3_ NCs. e) A high‐resolution TEM (HRTEM) image of a single CsSn_0.6_Ge_0.4_I_3_ nanocube. f) Fast Fourier transform (FFT) analysis of the HRTEM image. e) Narrow size distribution histogram of the particles.

Based on the previous hot‐injection methods to produce Sn‐based PNCs,[^30^, ^31^] we developed a modified reaction route for the synthesis of CsSn_*x*_Ge_1−*x*_I_3_ NCs. An equal molar ratio of SnI_2_ and GeI_2_ has been utilized to target the half replacement of Sn atoms by Ge atoms in the expected stoichiometry (namely, *x*=0.5). We found that the injection temperature is crucial to form stable SnGe‐PNCs at least before the purification process. The temperature should be above 240 °C. After the injection of Cs‐oleate into the precursor of Sn/Ge halide source, a sudden color change from transparent orange to dark brown occurred (see the appearance of CsSn_*x*_Ge_1−*x*_I_3_ NCs suspension in the side photo in Figure 1 a) and no further color change was observed after 3 s reaction time, indicating that the formation of SnGe‐PNCs features the extremely swift reaction kinetics, similarly as in the case of conventional Pb‐based PNCs.^[32]^ The detailed synthetic route for SnGe‐PNCs and the reference Sn‐PNCs has been described in Supporting Information.

To examine the crystal structure of as‐synthesized PNCs, X‐ray diffraction (XRD) patterns for PNCs in film state have been measured. Figure 1 a shows the comparison of XRD patterns for CsSn_*x*_Ge_1−*x*_I_3_ NCs and reference CsSnI_3_ NCs with the complete assignment of all the featured peaks corresponding to the halide perovskite structure.[^31^, ^33^] The XRD scan of reference Sn‐PNCs matches well with JCPDS reference 00‐043‐1162 from the ICDD database (Supporting Information, Figure S1a), indicating the formation of an orthorhombic crystallographic phase that belongs to the space group *Pnma* (62), which is consistent with previous literature findings for similar Sn‐based PNCs.^[31]^ It is noted that the XRD pattern of the newly synthesized SnGe‐PNCs shows slight shifts in the peak positions compared to that of the reference, that is, 14.2° to 14.5° at (110) and 29.0° to 29.2° at (220) (see inset in Figure 1 a), suggesting a slight change in the size of the unit cell dimension. This change is attributed to a partial exchange of the large Sn atoms with relatively small Ge atoms, resulting in the shrinkage of the unit cell and an upward shift in the 2*θ* angles. Therefore, we similarly assign the structure of as‐formed SnGe‐PNCs with orthorhombic crystallographic phase (Figure 1 b) as in the case of the reference. The surface compositions of CsSn_*x*_Ge_1−*x*_I_3_ NCs and reference CsSnI_3_ NCs in film state were analyzed by X‐ray photoelectron spectroscopy (XPS) measurements (Figure 1 c). The concentration of iodine should be considered as a lower estimate because of the X‐ray and electron beam induced desorption of I during XPS and EDS measurements.^[34]^ CsSn_*x*_Ge_1−*x*_I_3_ NCs had similar surface concentrations for Sn and Ge that support the successful substitution of Sn with Ge (Supporting Information, Table S1). Only one chemical state was resolved on CsSn_*x*_Ge_1−*x*_I_3_ NCs for Cs (Cs 4d_5/2_ at 75.2 eV), Sn (Sn 4d_5/2_ at 25.2 eV), Ge (Ge 3d_5/2_ at 31.6 eV), and I (I 4d_5/2_ at 48.8 eV), which can be attributed to Cs^+^, Sn^2+^, Ge^2+^, and I^−^, respectively, in accordance with the Cs^+^(Sn/Ge)^2+^I_3_^1−^ perovskite structure.[^29^, ^35^] The thorough XPS analysis confirms the stoichiometry of the PNCs films at the surface (that is, within the XPS information depth of 11 nm), to be CsSnI_3_ and CsSn_0.5_Ge_0.5_I_3_, with minor deviations (Supporting Information, Table S1). To further verify the stoichiometry of PNCs in bulk, we conducted energy‐dispersive X‐ray spectroscopy (EDS) measurements for the PNCs films. The EDS layered images SnGe‐PNCs (Supporting Information, Figure S2) for the compositional elements show the even elemental distribution on the surface of PNCs film. The EDS analysis elucidates the stoichiometry of SnGe‐PNCs bulk film (Supporting Information, Table S1). Interestingly, the elemental ratio between Sn and Ge turns to 2:1, meaning that the actual replacement proportion of Sn atoms by Ge atoms is nearly 40 % while considering the results of XPS data for the EDS analysis. We thus specify *x*=0.6 in the stoichiometry, outlining CsSn_0.6_Ge_0.4_I_3_ as the final composition. The Ge amount dependent synthesis and properties of SnGe‐PNCs will be investigated in a separate work. It is noted that the ratio of Cs:I in the bulk case is less than the nominal value (for example, 1:3; Supporting Information, Table S1), which can be attributed to the small ratio (1:4) of the injected volume (Cs‐oleate) and that of SnI_2_/GeI_2_ precursor, creating the halide‐rich environment to form polyhedral [SnI_6_]^4−^ as nucleus for the growth of nanocrystals.^[36]^

The morphology of as‐synthesized CsSn_0.6_Ge_0.4_I_3_ NCs was investigated using transmission electron microscopy (TEM). Figure 1 d reveals that the nanoparticles have a cubic shape with an average square diameter of about 9.4 nm for CsSn_0.6_Ge_0.4_I_3_ NCs, which is slightly larger than that (ca. 8.3 nm) of CsSnI_3_ NCs (Supporting Information, Figure S3a). Since the synthesis conditions were identical for both PNCs, this may suggest that small Ge atoms can potentially assist large Sn atoms to de‐focus over a longer growth duration more effectively than pure Sn‐based ones according to a kinetics model for the growth of Pb‐based PNCs,^[37]^ hence resulting in the bigger size of SnGe‐PNCs. Based on the HRTEM image of a single CsSn_0.6_Ge_0.4_I_3_ nanocube (Figure 1 e), the lattice distance is 0.317 nm that corresponds to (220) facets, highly consistent with the calculated lattice spacing of 3.17 Å from the Fast Fourier Transform (FFT) analysis (Figure 1 f) of the HRTEM image. This, together with the previous XRD data, further supports an orthorhombic crystal structure.^[31]^ Narrow size distributions have been observed with full widths at half‐maximum (FWHM) of 1.9 nm (Figure 1 g) and 1.4 nm (Supporting Information, Figure S3b) for SnGe‐PNCs and Sn‐PNCs, respectively. This suggests that our modified hot‐injection synthetic route can precisely control the nucleation and growth of nanocubes.

### Optical Properties of CsSn_0.6_Ge_0.4_I_3_ NCs

**Figure 2:**
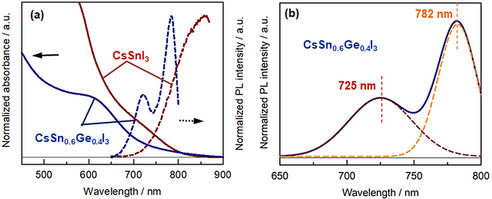
a) Absorption spectra (solid lines) and photoluminescence (PL) spectra (dashed lines) of CsSnI_3_ NCs and CsSn_0.6_Ge_0.4_I_3_ NCs in suspension, respectively. b) Deconvolution of the PL profile of CsSn_0.6_Ge_0.4_I_3_ NCs.

The optical properties of as‐synthesized CsSn_0.6_Ge_0.4_I_3_ NCs were investigated and compared to those of reference CsSnI_3_ NCs at room temperature in ambient conditions. The comparison of absorption and photoluminescence (PL) spectra of both PNCs in suspension is presented in Figure 2 a. A clear blue shift has been observed for both absorption onset and PL peak from CsSnI_3_ NCs to CsSn_0.6_Ge_0.4_I_3_ NCs due to the wider *E_g_* obtained upon the introduction of Ge in the Sn‐based nanocrystal structure. The PL spectrum of CsSn_0.6_Ge_0.4_I_3_ NCs shows a main peak at around 780 nm (1.6 eV) with a FWHM of 65 nm, while a small PL sub‐peak at around 725 nm has been detected that was not observed for the case of reference CsSnI_3_ NCs. We deconvoluted the emission spectrum with two contributed bands showing respective Gaussian peaks in Figure 2 b, centered at 725 and 782 nm.^[38]^Interestingly, we have found that a small amount of SnGe‐based nanorods formed together with the nanocubes. The nanorods have an average diameter of about 4.5 nm and a mean length of about 50 nm (see the corresponding TEM image in the Supporting Information, Figure S4a). This finding explains the origin of the 725 nm emission. The band gap of perovskite nanorods is closely related to their diameter or width.[^39^, ^40^] The small size of the Sn‐Ge nanorods (diameter of 4.5 nm) may induce a quantum confinement effect compared to the big SnGe‐nanocubes (diameter of 9.4 nm), with a corresponding shift towards a higher band gap when the size of PNCs is less than their Bohr radius.^[39]^ This phenomenon has not been observed for reference Sn‐PNCs, since in that case a change in the morphology has not been detected, as for the case of the mixed Sn^2+^ and Ge^2+^ cations. This point will be more thoroughly investigated in a future work. The absorption and PL spectra in film state for both PNCs (Supporting Information, Figure S4b) are nearly identical to those in suspensions, suggesting that no morphological change occurs during the formation of the PNC bulk film (see the inset photo of CsSn_0.6_Ge_0.4_I_3_ NCs film in the Supporting Information, Figure S4b). The PL quantum yields (PLQYs) were sequentially measured as 0.95 % and 0.16 % for CsSn_0.6_Ge_0.4_I_3_ NCs and reference CsSnI_3_ NCs, respectively. Although the absolute values of PLQYs are low compared to the lead analogues, a dramatic increase in the PLQY of CsSn_0.6_Ge_0.4_I_3_ NCs has been achieved compared to that of the Ge‐free counterpart. Since the dominant defects in Sn‐based PNCs are interstitial, and Sn displays low defect formation energy in terms of the metal terminate mechanism,^[41]^ the involvement of Ge effectively fills in Sn vacancies and reduces the number of traps, resulting in the enhanced PLQY.

### Photophysical Properties of CsSn_0.6_Ge_0.4_I_3_ NCs

**Figure 3:**
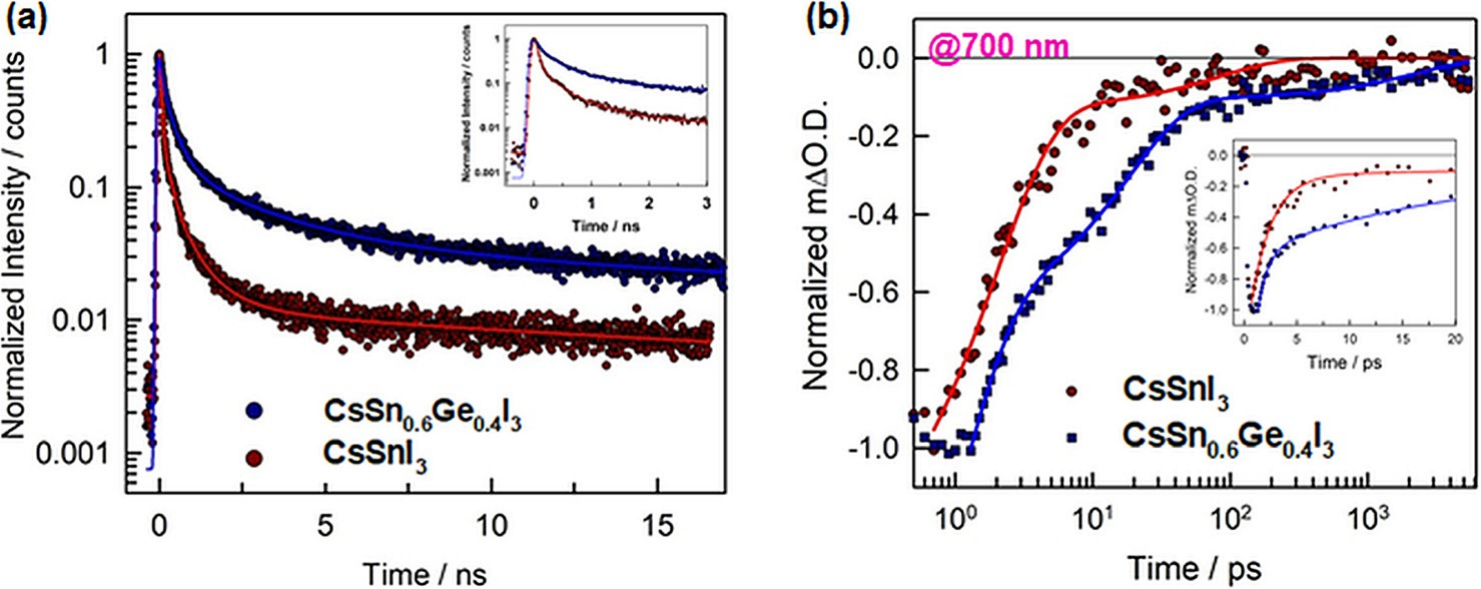
a) Time‐correlated single photon counting (TCSPC) decays of CsSnI_3_ NCs and CsSn_0.6_Ge_0.4_I_3_ NCs in suspension (the inset shows magnified decays in early timescale), excited at 405 nm. Solid lines present the fitting results by Poisson statistics model. c) Ultrafast transient absorption (TA) decays of the encapsulated CsSnI_3_ NCs and CsSn_0.6_Ge_0.4_I_3_ NCs films excited upon a 500 nm pump light with an excitation energy intensity of 10 μJ cm^−2^. Solid lines show the fitting results with a bi‐exponential function.

We then turn to assess the PL dynamics of as‐synthesized nanocrystals by conducting the time‐correlated single photon counting (TCSPC) measurements in the pico‐ and nanosecond regime. The PL decays of nanocubes in suspension are presented in Figure 3 a. Apparently, the emission decay of CsSnI_3_ NCs is faster than that of CsSn_0.6_Ge_0.4_I_3_ NCs. Poisson statistics of defects distribution in PNCs have been employed to model decays and fit the experimental data (see the detail of the Poisson model in Supporting Information).[^42^, ^43^] It was found that two types of defects need to be assumed to obtain a reasonable data fit. The fitting results are shown in the Supporting Information, Table S2, where *τ* is the quenching time constant in a nanocrystal having exactly one defect and *c* is the relative defect concentration, or the average number of defects per nanocrystal. The observation of short PL lifetime for CsSnI_3_ NCs makes a good agreement with previously reported fast decays for Sn‐based nanocrystals within several hundreds of picoseconds.[^30^, ^31^] In contrast to the reference, the quenching time constants of CsSn_0.6_Ge_0.4_I_3_ NCs have been extended by a factor of 4 to 490 and 4200 ps, respectively. At the same time the relative defect concentrations are also lower for CsSn_0.6_Ge_0.4_I_3_ NCs, *c*_1_=2.0, *c*_2_=1.7, vs. *c*_1_=2.3, *c*_2_=3.3 of CsSnI_3_ NCs (Table S2). This supports our hypothesis of Ge filling the Sn vacancies, which has the two side effects of 1) lowering the density of defects and 2) the less detrimental effect of the defects left, that is, slower quenching. Furthermore, it is noted that the PL decay lifetimes of both PNCs in film (Supporting Information, Figure S5) state are similar to those of their suspensions, suggesting that the defect distribution for both PNCs is less influenced by their variable phases.

To further investigate the influence of Ge replacement on the charge carrier dynamics and the nonradiative processes, we also carried out the ultrafast transient absorption (TA) spectroscopy on the encapsulated CsSnI_3_ NCs and CsSn_0.6_Ge_0.4_I_3_ NCs films on glass, respectively. The TA spectra of the CsSnI_3_ NCs film excited upon a 500 nm pump light after different time delays are shown in the Supporting Information, Figure S6a. The overall TA spectra are dominated by one distinct bleach signal with a peak at 700 nm, which are corresponding to the first (660–800 nm) excitonic absorption band, in a good agreement with previously reported assignments for CsSnI_3_ QDs.^[44]^ It is noted that there is a positive signal appearing with a peak at 600 nm, likely attributed to the overlap of the bleach with the photoinduced absorption (PIA) between 570 and 650 nm. The comparative TA spectra of CsSn_0.6_Ge_0.4_I_3_ NCs film measured upon the identical condition as for the case of reference Sn‐PNCs film are shown in the Supporting Information, Figure S6b. Interestingly, a second bleached band appears at 750 nm in addition to the main bleached band below 700 nm. We conclude that the second bleach arises from the small amount of nanorods mixed into the sample. After assigning the origins in the TA spectra of both PNCs films, we now turn to assess the TA kinetics. A comparison of TA decays for CsSnI_3_ NCs and CsSn_0.6_Ge_0.4_I_3_ NCs films, monitored at 770 nm in early timescale are shown in the Supporting Information, Figure S6c. It is clearly observed that both TA profiles instantly rise within the instrument time (ca. 0.2 ps), corresponding to the formation of hot carriers after excitation well above the band gap (500 nm pump light). The hot carriers then relax to the band edge within a cooling time of about 2.5 ps for both films, indicating that the partial replacement of Ge atoms in nanostructure does not make obvious effect on the hot carrier kinetics. We then compared the TA decays of both films monitored at 700 nm (Figure 3 b) to evaluate the dynamics of bleaching recovery. The TA decay curves can be well fitted using a bi‐exponential function: ΔO.D.=*A*_1_×exp(−*t*/*τ*_1_) + *A*_2_×exp(−*t*/*τ*_2_), which provides two exponential decay constants for both films (summarized in the Supporting Information, Table S3). As discussed earlier, Ge atoms can effectively fill the high density of Sn vacancies, reducing the shallow traps and leading to a much longer average lifetime (*τ*_avg_=259.4 ps) compared to that of reference CsSnI_3_ NCs film (*τ*_avg_=10.1 ps), which is highly consistent with the trend of TRPL data in the Supporting Information, Table S2. This further suggests that, compared to reference CsSnI_3_ NCs, the CsSn_0.6_Ge_0.4_I_3_ NCs possess the extended excited‐state lifetime that allows efficient charge transfer, which is beneficial for the device performance.

### Enhanced Performance of CsSn_0.6_Ge_0.4_I_3_ NCs‐based Solar Cells

**Figure 4:**
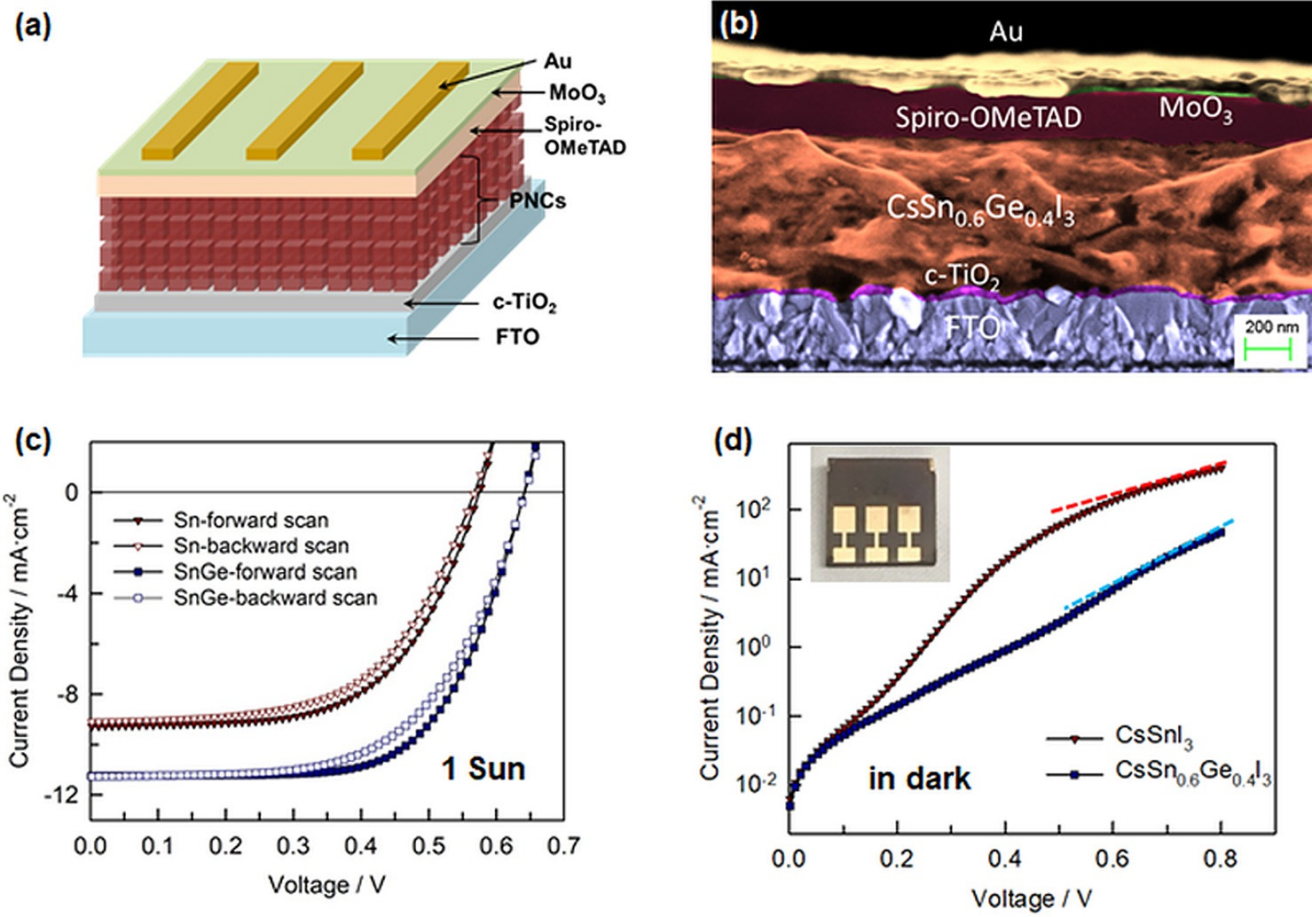
a) Architecture of *n‐i‐p* PNCs based solar cells in planar structure studied in this work. b) Cross‐sectional SEM image of CsSn_0.6_Ge_0.4_I_3_ NCs‐based solar cell, observed at 3 kV. c),d) *J*–*V* curves of the champion Sn‐PNCs and SnGe‐PNCs based solar cells (c) under 1 sun illumination (forward and backward scans with a scan rate of 10 mV s^−1^) and (d) in dark condition (forward scan) while dash line shows the fitting to extract series resistance. The inset picture shows the appearance of a typical CsSn_0.6_Ge_0.4_I_3_ NCs‐based solar cell.

The improved photophysical properties of CsSn_0.6_Ge_0.4_I_3_ NCs compared to the case of reference CsSnI_3_ NCs has a direct effect on the performance of corresponding PSCs. To confirm this, we incorporated both types of nanocubes in standard *n‐i*–*p* photovoltaic architectures. Owing to the reported high charge diffusion coefficient (>1.3 cm^2^ s^−1^) for typical bulk CsSnI_3_ crystals, a planar structure has been adopted for this type of perovskite.[^45^, ^46^] The full PSC structure is FTO/c‐TiO_2_/PNCs/spiro‐OMeTAD/MoO_3_/Au, as illustrated in Figure 4 a. The devices consisted of circa 550 nm thick PNCs layer (based on the cross‐sectional SEM image of a typical CsSn_0.6_Ge_0.4_I_3_ NCs‐based solar cell, Figure 4 b), fabricated in a nitrogen‐filled glovebox. The current density (*J*)‐voltage (*V*) curves of the best‐performing (champion) devices based on SnGe‐PNCs and reference Sn‐PNCs, recorded under standard 1 Sun condition (100 mW cm^−2^ AM 1.5 G illumination) and in dark condition, are depicted in Figure 4 c,d, respectively. Interestingly, the *J*–*V* curves of both PNCs‐based devices display a negligible hysteresis effect between forward and backward scans (Figure 4 c). This suggests that ion migration under an electric field has a nearly negligible influence on the photocurrent flow within the perovskite nanocrystals layer. This, in turn, may lead to a more stable power output of the corresponding PNCs devices compared to that of bulk crystals‐based devices, such as the popular tri‐cation perovskite solar cells in *n‐i‐p* structure.^[2]^ The averaged photovoltaic performances (16 devices for each structure) along with the standard deviation, together with the photovoltaic parameters of the champion cells, are summarized in Table 1. The high reproducibility of our results is demonstrated by the small standard deviations. The CsSn_0.6_Ge_0.4_I_3_ NCs lead to PSCs with a remarkable 58 % performance enhancement (average PCE=4.1 %) compared to devices based on reference CsSnI_3_ NCs (Table 1) in this work, resulting in the highest PCE of 4.9 % for the champion cell (shown in the inset photo in Figure 4 d).

**Table 1:** Summary of PNCs based solar cells performance.^[a]^

PNCs	*J*_SC_ [mA cm^−2^]	*V*_OC_ [V]	FF	PCE [%]	*R*_S_ [Ω cm^2^]	*R*_SH_ [kΩ cm^2^]
Sn‐PNCs	8.6±0.8 (9.4)^[b]^	0.54±0.04 (0.58)	0.54±0.03 (0.57)	2.7±0.4 (3.1)	23.3±1.5 (21.8)	3.5±0.3 (3.8)
SnGe‐PNCs	10.9±0.9 (11.8)	0.61±0.03 (0.65)	0.60±0.03 (0.64)	4.1±0.8 (4.9)	13.4±1.1 (12.3)	6.3±0.4 (6.7)

[a] The data are from the forward scans. [b] Values in the brackets refer to the photovoltaic parameters of the champion cells.

The improvement in the PCE is clearly determined by the increase in all device parameters, that is, *J*_SC_, *V*_OC_, and FF. We have extracted the series (*R*_S_) and shunt (*R*_SH_) resistances of the devices, according to the 1‐diode model,^[47]^ from the *J*‐*V* curves scanned in dark with the axis of current density plotted in logarithmic scale (Figure 4 c and Table 1). Both *R*_SH_ and *R*_S_ affect the FF, with lower *R*_S_ and higher *R*_SH_ being required to increase the FF. The extracted resistances in Table 1 confirm this trend since CsSn_0.6_Ge_0.4_I_3_ NCs‐based cells have lower *R*_S_ and higher *R*_SH_ (nearly twice) than that of the CsSnI_3_ NCs‐based cells. In addition, the excited‐state lifetime (ca. 4 ns) of CsSn_0.6_Ge_0.4_I_3_ NCs clarified from the TCSPC and TA measurements, which is nearly three times longer than that (ca. 1.5 ns) of CsSnI_3_ NCs, in turn leads to a more effective charge separation at the interface between PNCs film and charge‐transporting layers (TiO_2_ or spiro‐OMeTAD).^[48]^ This can be one key reason for the enhanced *J*_SC_ and *FF* in CsSn_0.6_Ge_0.4_I_3_ NCs‐based cells. The above‐demonstrated involvement of Ge in reducing the Sn vacancies (that function as trapping sites) suggests that CsSn_0.6_Ge_0.4_I_3_ NCs‐based active layer possesses much fewer defect sites compared to the CsSnI_3_ NCs film, leading to the hindering of charge recombination within PNCs film beneficial to the *V*_OC_.[^49^, ^50^] Furthermore, we investigated the surface morphology of PNCs films by taking SEM topographic imaging for CsSn_0.6_Ge_0.4_I_3_ NCs (in Figure S7a) and CsSnI_3_ NCs (Supporting Information, Figure S7b). It is noted that the surface of CsSn_0.6_Ge_0.4_I_3_ NCs film is much smoother and denser (see inset image in the Supporting Information, Figure S7a) than that of CsSnI_3_ film. Probably, upon the involvement of Ge, Sn phase aggregation can be avoided, supporting the possibility of suppressed interfacial charge recombination.

### Improved Stability of CsSn_0.6_Ge_0.4_I_3_ NCs

**Figure 5:**
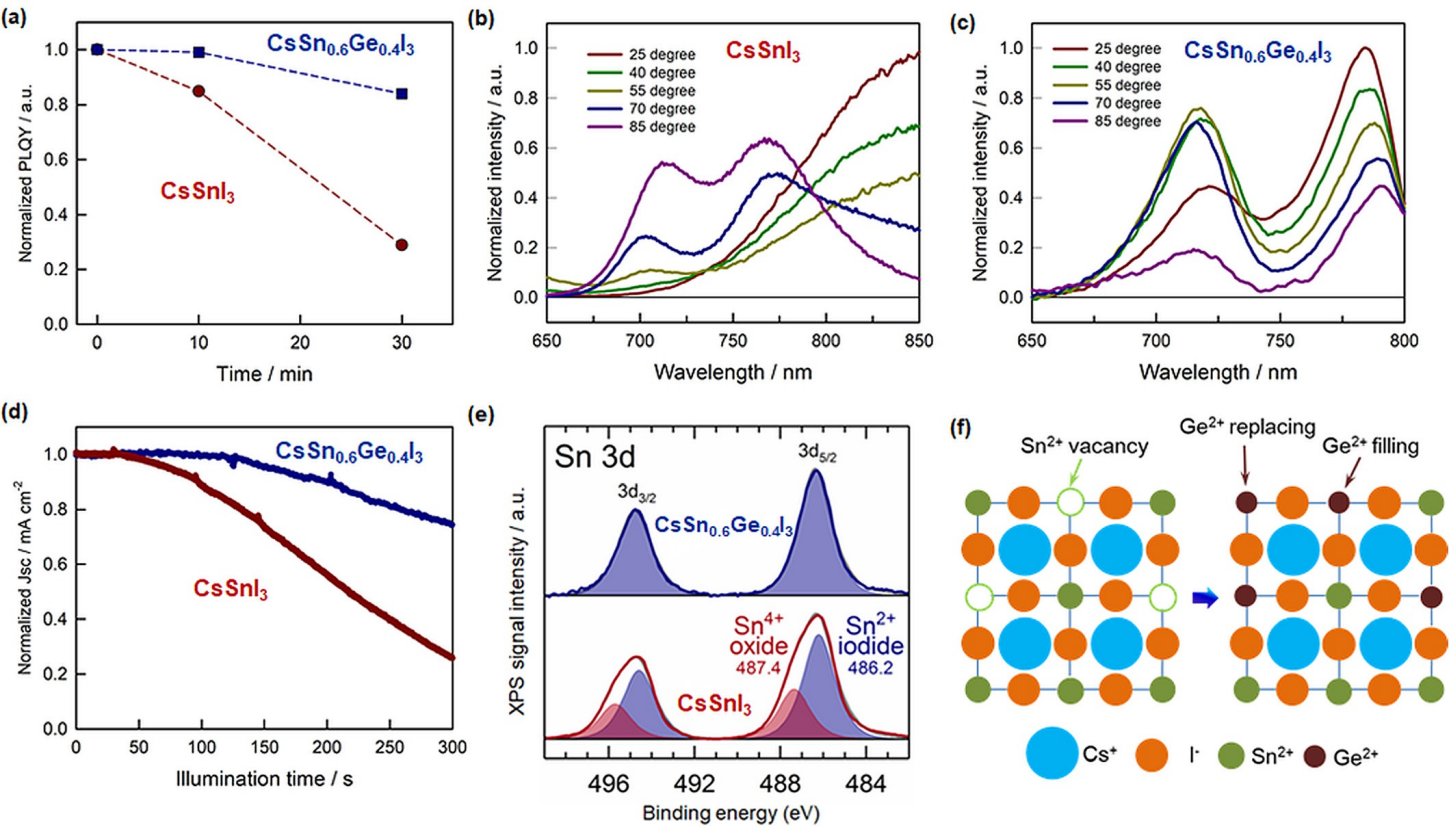
a) Time‐dependent PLQYs of CsSnI_3_ NCs and CsSn_0.6_Ge_0.4_I_3_ NCs in suspension under ambient condition. Temperature dependent PL spectrum of b) CsSnI_3_ NCs and c) CsSn_0.6_Ge_0.4_I_3_ NCs in suspension under ambient condition. d) *J_SC_* evolution of typical unencapsulated CsSnI_3_ NCs and CsSn_0.6_Ge_0.4_I_3_ NCs‐based PSCs in continuous operation under 1 sun illumination in air. The sampling time is 100 ms. e) XPS analysis of Sn 3d transition for CsSnI_3_ NCs and CsSn_0.6_Ge_0.4_I_3_ NCs after 10 min exposure to air atmosphere. f) Scheme of the Ge filling effect in the Sn vacancies and the replacement of Sn atoms by Ge atoms in the nanocrystal structure.

Finally, the effect of Ge on the stability of the corresponding PNCs is thoroughly studied. Figure 5 a shows the comparison between the time‐dependent PLQYs of CsSnI_3_ NCs and CsSn_0.6_Ge_0.4_I_3_ NCs in suspension in air (25 °C and 50 % RH). While the PLQY of CsSnI_3_ NCs swiftly decreased to below 30 % of its initial value after 30 min (Supporting Information, Figure S8a), that of CsSn_0.6_Ge_0.4_I_3_ NCs was retained to above 80 % of the original PLQY in the same time span (Supporting Information, Figure S8b). This suggests that upon the protection of Ge atoms in the Sn‐based perovskite against the oxidization, the optical properties of PNCs can be effectively maintained. Figure S9 presents the comparison of XRD patterns from unencapsulated reference CsSnI_3_ NCs (Supporting Information, Figure S9a) and CsSn_0.6_Ge_0.4_I_3_ NCs (Supporting Information, Figure S9b) films exposed for 10, 20, and 30 min to ambient atmosphere. The relative intensity of the main XRD peak can sustain above 75 % of its initial value for the case of SnGe‐PNCs after 30 min exposure while that of Sn‐PNCs has dropped below 45 % of its corresponding initial intensity, consistent with the enhancement of optical stability upon the introduction of Ge. Moreover, we measured the temperature‐dependent PL spectrum (Figure 5 b,c) for both PNCs under ambient conditions to clarify the thermal stability influenced by the involvement of Ge in the perovskite structure. Interestingly, upon temperatures above 55 °C, the PL spectrum of CsSnI_3_ NCs exhibited a clear decrease in the PL intensity with a distinct blue‐shift and an additional sub‐peak at about 710 nm, indicating a sign of the phase transition or degradation, that is, the formation of Sn^4+^. On the contrary, the PL spectrum of CsSn_0.6_Ge_0.4_I_3_ NCs retained its shape feature when temperature increased, although the peak PL intensity at 782 nm (corresponding to nanocubes) decreased, as commonly observed for other types of perovskite nanocrystals.[^51^, ^52^] It is interestingly noted that the sub‐peak PL intensity at 725 nm (corresponding to nanorods) effectively increased up to 70 °C and then decreased with the increasing temperature (85 °C), indicating that some temperature‐dependent morphological transition from nanocubes to nanorods may occur which will be investigated in detail in a separate work. The overall study of PL stability suggests that Ge atoms can effectively stabilize the nanocrystal structures upon the thermal treatment. We then studied the stability of the corresponding photovoltaic devices by measuring the change in the short circuit current density (*J*_SC_) under continuous 1‐sun illumination in air. Figure 5 d compares the decrease in *J*_SC_ upon unencapsulated cells illumination for both CsSnI_3_ NCs‐ and CsSn_0.6_Ge_0.4_I_3_ NCs‐based devices. As predicted, *J*_SC_ of pristine Sn‐PNCs cells decreased by more than 75 % of its initial value after 300 s of continuous illumination. On the other hand, SnGe‐PNCs based devices can still retain more than 80 % of initial *J*_SC_, which is consistent with the variation trend of the time‐dependent PLQYs of both PNCs. Hence, CsSn_0.6_Ge_0.4_I_3_ NCs exhibit significantly improved ambient, thermal, and photostability compared to CsSnI_3_ NCs.

To investigate the underlying reason for the improved stability of CsSn_0.6_Ge_0.4_I_3_ NCs, we can further refer to the XPS analysis in Figure 5 e. Clearly, only one chemical state containing Sn could be extracted for CsSn_0.6_Ge_0.4_I_3_ NCs (Sn 3d_5/2_ at 486.2 eV) after 10 mins exposure to air, that is, during the sample preparation before the XPS measurement. In contrast, within the same time frame, a second chemical state was clearly observed for CsSnI_3_ NCs related to Sn 3d transition: a second pair of doublet peaks (Sn 3d_5/2_ at 487.4 eV) is found in addition to the Sn^2+^ iodide 3d_5/2_ at 486.2 eV. The high binding energy component corresponds to Sn^4+^ oxide.[^53^, ^54^, ^55^] This was also confirmed by the O 1s metal oxide component at 530.7 eV, which has been observed only for the case of pure Sn‐PNCs. Furthermore, we have conducted the XPS analysis on CsSn_0.6_Ge_0.4_I_3_ NCs after 24 hours of exposure to air (Supporting Information, Figure S10). Upon the long‐time exposure to air, Sn 3d/4d peaks are shifted to higher binding energies, indicating the oxidation of Sn^2+^ cations into Sn^4+^ state. Yet, the Ge 3d peak has shown no obvious change with the fixation of most Ge^2+^ cations in the perovskite structure. Interestingly, the fast formation of Ge^4+^ oxide that was recently observed by Chen et al. on bulk perovskite with a similar elemental composition (bulk CsSn_0.5_Ge_0.5_I_3_) but prepared using a melt‐crystallization method has not been observed in our case.^[29]^ Furthermore, the enhanced stability of CsSn_0.6_Ge_0.4_I_3_ NCs is in agreement with two factors demonstrated in the previous simulation results: 1) the Goldschmidt tolerance factor (*t*) has been reported for the mixed Sn‐Ge halide perovskite in comparison to Ge‐free counterpart,^[56]^ that is, *t* (MASn_0.5_Ge_0.5_I_3_)=0.90, and *t* (MASnI_3_)=0.84, indicating that the involvement of Ge atoms in Sn‐based perovskite structure is beneficial for the structural stability; 2) the activation barrier (0.23 eV) towards water or oxygen penetration of mixed Sn‐Ge halide perovskite (RbSn_0.5_Ge_0.5_I_3_) has been reported as remarkably higher than that of pure Sn perovskite (0.17 eV for MASnI_3_)^[56]^ or even of Pb‐based perovskite (0.09 eV for MAPbI_3_),^[27]^ suggesting that the terminated Ge atoms can effectively protect the inner Sn atoms, making them less susceptible to oxidization upon the replacement and filling effect from Ge atoms (see the proposal in Figure 5 f).

## Conclusion

We have shown the first‐ever synthesis of CsSn_0.6_Ge_0.4_I_3_ nanocrystals with a narrow size distribution. The PLQY of Sn‐Ge based NCs is close to 1 %, which has enhanced nearly one order magnitude than that of conventional pure Sn‐based PNCs. The photophysical properties of CsSn_0.6_Ge_0.4_I_3_ NCs are dramatically enhanced compared to those of pure Sn‐PNCs, mainly attributed to the prolonged excited‐state lifetime, as confirmed by both transient photoluminescence and transient absorption measurements. We assign the improvement of both optical and photophysical properties of CsSn_0.6_Ge_0.4_I_3_ NCs to the reduced number of Sn vacancies upon their filling with Ge in the nanostructure. Vacancies act as trapping sites and alternative nonradiative recombination pathways. The overall improved charge transfer dynamics for CsSn_0.6_Ge_0.4_I_3_ NCs system, that is, efficient charge separation and suppressed charge recombination, is the key reason for the significant enhancement of solar cells performance when CsSn_0.6_Ge_0.4_I_3_ NCs are used as the light absorber instead of CsSnI_3_ NCs. The highest PCE of CsSn_0.6_Ge_0.4_I_3_ NCs‐based solar cells in this work, 4.9 %, is one of the highest values reported for all‐inorganic lead‐free PNCs solar cells. The introduction of Ge is highly beneficial for ambient, thermal, and photostability, due to the effective protection of Ge^2+^ against Sn^2+^ oxidization. We believe that, upon optimization (for example, by identifying the most suitable selective contacts matching the CsSn_0.6_Ge_0.4_I_3_ NCs energy levels), the solar cells based on SnGe‐PNCs can outperform current generation of lead‐free PNCs based solar cells with the additional benefit of outstanding stability, hence bringing PNCs‐photovoltaics a step closer to their commercialization.

## Conflict of interest

The authors declare no conflict of interest.

## Supporting information

As a service to our authors and readers, this journal provides supporting information supplied by the authors. Such materials are peer reviewed and may be re‐organized for online delivery, but are not copy‐edited or typeset. Technical support issues arising from supporting information (other than missing files) should be addressed to the authors.

Supplementary
