# Supplementary material for: B‐Site Co‐Alloying with Germanium Improves the Efficiency and Stability of All‐Inorganic Tin‐Based Perovskite Nanocrystal Solar Cells
Source: Angew Chem Int Ed Engl. 2020 Sep 25;59(49):22117–25. doi: 10.1002/anie.202008724 (PMC7756719; doi:10.1002/anie.202008724)
Supplement: Supplementary file 1 — Supplementary [file ANIE-59-22117-s001.pdf]

Angewandte Chemie
Eine Zeitschrift der Gesellschaft Deutscher Chemiker

Supporting Information

B-Site Co-Alloying with Germanium Improves the Efficiency and Stability of All-Inorganic Tin-Based Perovskite Nanocrystal Solar Cells

*Maning Liu, Hannu Pasanen, Harri Ali-Löytty, Arto Hiltunen, Kimmo Lahtonen, Syeda Qudsia, Jan-Henrik Smått, Mika Valden, Nikolai V. Tkachenko, and Paola Vivo**

anie_202008724_sm_miscellaneous_information.pdf

SUPPORTING INFORMATION

Table of contents

1. Experimental procedures 2

1.1 Chemicals 2

1.2 Preparation of Cs-oleate 2

1.3 Synthesis of $CsSn_{0.6}Ge_{0.4}I_3$ NCs 2

1.4 Device fabrication in *n-i-p* planar structure 2

1.5 Characterizations 2

2. Results 3

2.1 Structure of reference Sn-PNCs 3

2.2 EDS analysis of PNCs 3

2.3 Morphology and size distribution of reference $CsSnI_3$ NCs 4

2.4 Variable morphologies of $CsSn_{0.6}Ge_{0.4}I_3$ NCs and optical properties of both PNCs in film state 4

2.5 TRPL decays of $CsSnI_3$ NCs and $CsSn_{0.6}Ge_{0.4}I_3$ NCs in film state 5

2.6 Analysis of Poisson model fitting for TRPL data 5

2.7 Transient absorption (TA) spectra and decays of $CsSnI_3$ NCs and $CsSn_{0.6}Ge_{0.4}I_3$ NCs films 6

2.8 Fitting results of the TA decays of $CsSnI_3$ NCs and $CsSn_{0.6}Ge_{0.4}I_3$ NCs films 7

2.9 Morphology study of PNCs-based films 7

2.10 Optical stability of $CsSnI_3$ NCs and $CsSn_{0.6}Ge_{0.4}I_3$ NCs 7

2.11 Time-dependent XRD patterns of PNCs 8

2.12 XPS analysis of aged $CsSn_{0.6}Ge_{0.4}I_3$ NCs 8

References 8

Author Contributions 8

1. Experimental procedures

1.1 Chemicals

Cs_2CO_3 (99.9%), GeI_2 (≥ 99.8%), octadecene (ODE, 90%), oleic acid (OA, 90%), oleylamine (OAm, technical grade, 70%), titanium diisopropoxide bis(acetylacetonate) (TDBA) 75 wt.% in isopropanol, bis(trifluoromethane)sulfonimide lithium salt (Li-TFSI, 99.95%), chlorobenzene (extra dry, 99.8%), acetonitrile (99.9%), and 4-tert-butyilpyridine (4-tBP) were purchased from Sigma-Aldrich. SnI2 (> 98%) was obtained from TCI Europe. Spiro-OMeTAD (99%) and tris[2-(1H-pyrazol-1-yl)-4-tert-butylpyridine]cobalt(III)tri[bis-(trifluoromethane) sulfonimide] (FK209 Co(III), >98%) were purchased from Lumtec and Dyenamo, respectively. All chemicals were used without further purification.

1.2 Preparation of Cs-oleate

Cs_2CO_3 (0.271 g) was loaded into a 50 mL three-neck flask along with octadecene (ODE, 10 mL) and oleic acid (1 mL), degassed under vacuum for 1 h at 120 °C, and then heated under Ar at the same temperature until all Cs_2CO_3 was dissolved by showing a transparent solution. Since Cs-oleate precipitates out of ODE at room temperature, it needs to be preheated (e.g. at 120 °C) before reaction.

1.3 Synthesis of $CsSn_{0.6}Ge_{0.4}I_3$ NCs

In a typical hot-injection synthesis, 0.253 g of SnI_2 (0.68 mmol) and 0.222 g of GeI_2 (0.68 mmol) (or only 0.507 g of SnI_2 to form $CsSnI_3$ NCs as reference), 10 mL of ODE, 1 mL of OA and 1 mL of OAm were loaded into a 50 mL three-neck flask and degassed under vacuum for 1 h at 120 °C. Then, the temperature was increased to 240 °C under Ar and 3.4 mL of as-prepared Cs-oleate solution (0.26 mmol) was swiftly injected under vigorous stirring. The color of the solution instantaneously turned from transparent orange (red for Sn-PNCs) to dark brown, exhibiting the formation of nanocrystals. The reaction was kept at the injection temperature for 30 s before a cold-water bath was set under the three-neck flask to quench the further growth of nanocrystals. No further color change was observed after about 3 s reaction time. The crude solution was then purified with an equal volume of 2-propanol to remove the unreacted precursors and by-products under nitrogen atmosphere. The resulting turbid mixture was centrifuged at 4500 rpm for 5 min, and after decantation the remaining PNC aggregates were re-dispersed in toluene before transferring into nitrogen-filled glovebox for device fabrication.

1.4 Device fabrication in *n-i-p* planar structure

FTO glass substrates (Greatcell Solar, TEC 15), 2 cm × 2 cm, were wet chemically etched with 2M HCl aqueous solution and zinc powder. The etched FTO substrates were then sonicated using an aqueous solution of Hellmanex III solution (2%), acetone and 2-propanol for 15 min in each step, successively. The substrates were then treated with UV-ozone for 15 min to remove organic residuals and increase hydrophilicity. A 30 nm thick compact TiO_2 layer (c-TiO_2) was deposited on the as-prepared patterned substrate by spray pyrolysis of 0.38 M titanium di-isopropoxide bis(acetylacetonate) solution in 2-propanol at 450 °C.[1][2] The films were then sintered at 450 °C for 1 h in air. The films were cooled down to 150 °C and directly transferred to a dry nitrogen glovebox. A ~550 nm thick PNCs active layer was then deposited on as-prepared c-TiO_2 layers by spin-coating a PNCs solution (120 mg/mL in toluene) at 1500 rpm for 30 s. The PNCs layer was then annealed at 100 °C for 20 min. After annealing, spiro-OMeTAD layer was spin-coated at 1800 rpm for 30 s. The spiro-OMeTAD solution was prepared by adding 36.2 mg spiro-OMeTAD to 1 ml chlorobenzene and 14.4 µl 4-tBP were stirred using a vortex mixer. Then 8.7 µl Li-TFSI solution and 14.5 µl FK209 pre-dissolved in acetonitrile were added to the spiro-OMeTAD solution with concentrations of 520 mg/ml and 300 mg/ml, respectively. Finally, a 5 nm thick MoO_3 interlayer and an 80 nm thick gold contact were thermally evaporated on top of the spiro-OMeTAD layer to form the back contact. Evaporation was conducted in high vacuum (6×10^{-6} mbar).

1.5 Characterizations

Electron microscopy images of the as-synthesized PNCs were obtained using a transmission electron microscope (TEM) operated at 200 kV (JEM-F200). Grazing incidence X-ray diffraction (XRD) was performed on samples spin-coated on glass substrates (Bruker AXS D8 Discover, CuKα radiation with λ = 1.5406 Å) and Eva software (Bruker AXS) was used for analysis of XRD data. X-ray photoelectron spectroscopy (XPS) was performed by employing an Al Kα X-ray source and Argus electron spectrometer (Omicron Nanotechnology GmbH). The surface composition was identified by analyzing core level spectra using CasaXPS software (Version 2.3.19 PR 1.0). The binding energy scale was calibrated according to C 1s (C–C/H) at 284.8 eV. Ultraviolet and visible absorption (UV−vis) spectra were recorded with a dual-beam grating Shimadzu UV-1800 absorption spectrometer. Photoluminescence (PL) spectra and quantum yield (QY) were obtained with a FLS1000 spectrofluorometer (Edinburgh Instruments, UK). The time-resolved PL (TRPL) decays were determined by using a time-correlated single photon counting (TCSPC) apparatus equipped with a Picoharp 300 controller and a PDL 800-B driver for excitation and a Hamamatsu R3809U-50 microchannel plate photomultiplier for detection in 90°

configuration. The instrument response time was 60 ps. Transient absorption (TA) measurements were conducted with a standard pump-probe system: the samples were excited by laser pulses at 400 nm (Libra F, Coherent Inc., coupled with Topas C, Light Conversion Ltd.) A white continuum generator (heavy water) was used to produce the probe beam. The TA responses were measured using an ExciPro TA spectrometer (CDP, Inc.) equipped with a CCD array. The SEM images of NCs films were obtained by field emission scanning electron microscope (FE-SEM, Carl Zeiss Ultra 55), operated at 3 kV. Energy dispersive X-ray spectroscopy (EDS) was performed using Zeiss UltraPlus FE-SEM electron microscope equipped with Oxford Instruments X-MaxN 80 EDS detector. Samples were drop casted on silicon substrates, and analysed using 15 kV acceleration voltage. The PNCs-based solar cells were characterized in air without encapsulating the devices. The active area of each cell was determined by the size of the aperture (0.2 cm^2), and accurately evaluated by an optical Dino-Lite AM4113ZTL micro-scope. The current density (*J*)-voltage (*V*) characteristics were recorded with a Keithley 4250 source-monitor unit, under air mass (AM) 1.5 simulated sunlight (100 mW/cm^2 irradiance). The illumination was generated through an AAA- solar simulator (Sciencetech Inc.), and calibrated using a silicon reference cell.

2. Results

2.1 Structure of reference Sn-PNCs

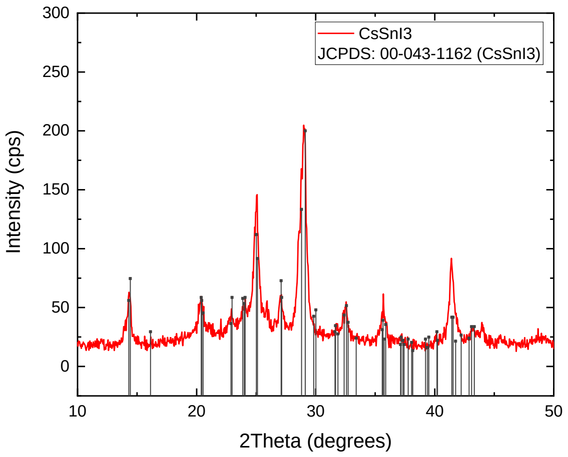

Figure S1. X-ray diffraction pattern of the as-prepared reference $CsSnI_3$ NCs, comparing with JCPDS reference 00-043-1162 from ICDD database.

2.2 EDS analysis of PNCs

Table S1. Elemental analysis for the resultant SnGe-PNCs and Sn-PNCs. The measured atomic concentration (at%) for each element is normalized with respect to iodine.

Analytical method	$CsSn_xGe_{1-x}I_3$				$CsSnI_3$		
	Cs	Sn	Ge	I	Cs	Sn	I
XPS (surface)	0.8	0.8	0.9	3.0	1.1	1.5	3.0
EDS (bulk)	0.4	0.6	0.3	3.0	0.7	1.0	3.0
Nominal	1	0.5	0.5	3.0	1.0	1.0	3.0

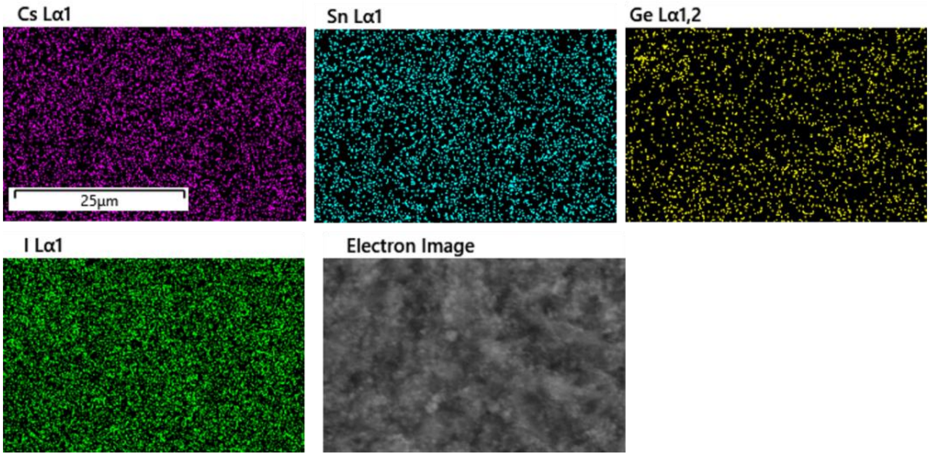

Figure S2. Energy-dispersive X-ray spectroscopy (EDS) layered images of Cs, Sn, Ge and I for $CsSn_xGe_{1-x}I_3$ NCs bulk film.

2.3 Morphology and size distribution of reference $CsSnI_3$ NCs

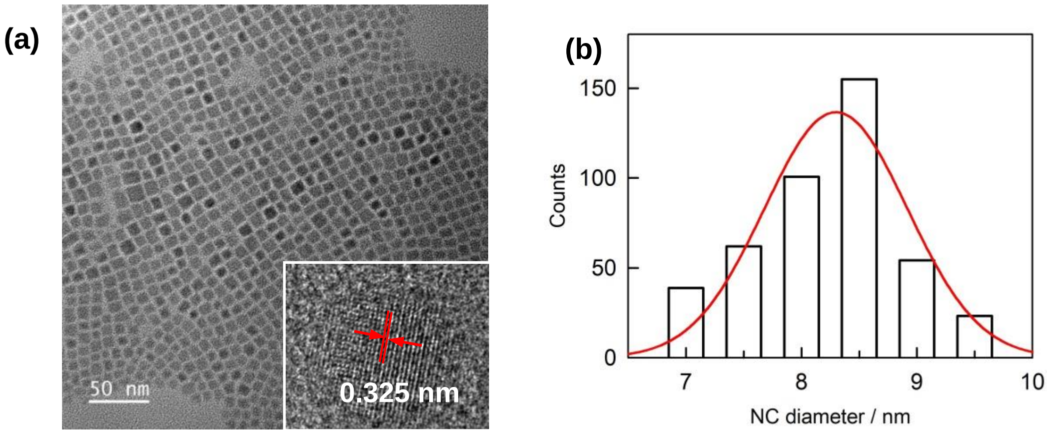

FigureS3. (a) Transmission electron microscopy (TEM) image of $CsSnI_3$ nanocubes; the inset presents a high-resolution TEM (HRTEM) image of a single $CsSnI_3$ nanocube, showing clear lattice fringes corresponding to (220) facets. (b) The narrow size distribution histogram of the particles, indicating an average diameter of 8.3 nm.

2.4 Variable morphologies of $CsSn_{0.6}Ge_{0.4}I_3$ NCs and optical properties of both PNCs in film state

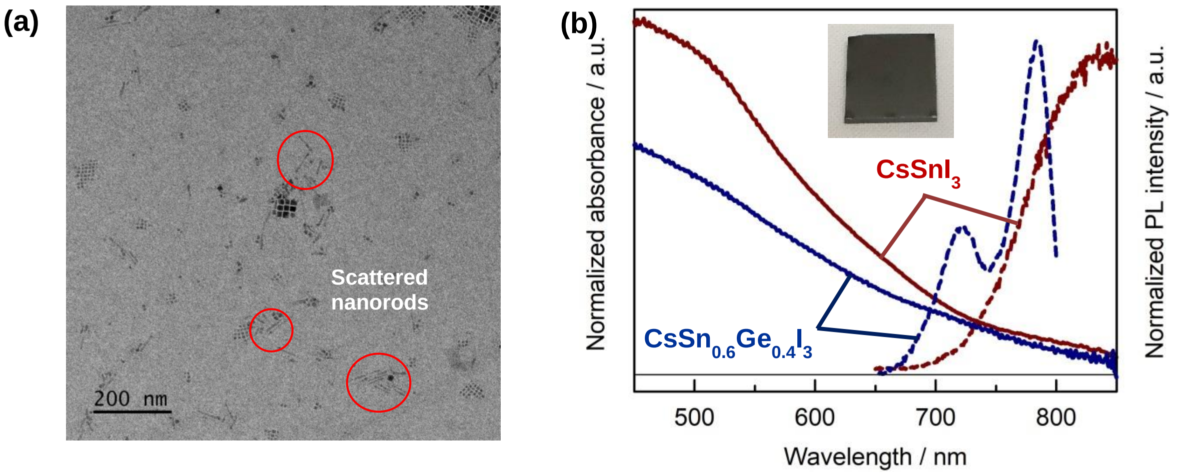

Figure S4. (a) TEM image of small amount of $CsSn_{0.6}Ge_{0.4}I_3$ NCs in nanorod shape highlighted in red circle. (b) Absorption spectra (solid lines) and photoluminescence (PL) spectra (dash lines) of $CsSnI_3$ NCs and $CsSn_{0.6}Ge_{0.4}I_3$ NCs in encapsulated film. The inset picture shows the appearance of $CsSn_{0.6}Ge_{0.4}I_3$ NCs film.

2.5 TRPL decays of $CsSnI_3$ NCs and $CsSn_{0.6}Ge_{0.4}I_3$ NCs in film state

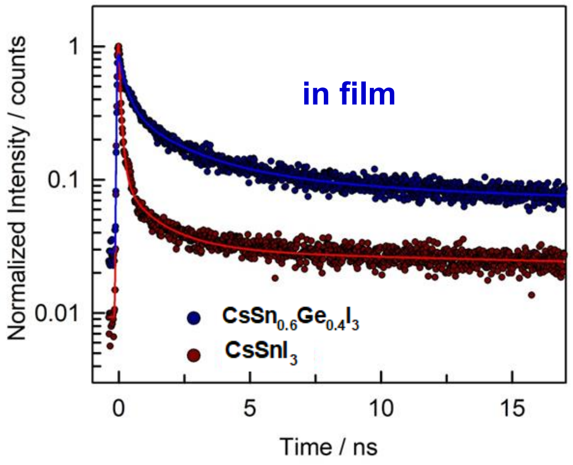

Figure S5. Time-correlated single photon counting (TCSPC) decays of $CsSnI_3$ NCs and $CsSn_{0.6}Ge_{0.4}I_3$ NCs in film state, excited at 405 nm, and monitored at 830 nm for Sn-PNCs and 780 nm for $CsSn_{0.6}Ge_{0.4}I_3$ NCs, respectively. Solid lines present the fitting results by Poisson statistics model.

2.6 Analysis of Poisson model fitting for TRPL data

Table S2. Summary of TRPL data for $CsSnI_3$ NCs and $CsSn_{0.6}Ge_{0.4}I_3$ NCs in both suspension and film state.

PNCs	In suspension				In film			
	τ_1 / ps	c_1[a]	τ_2 / ps	c_2	τ_1 / ps	c_1	τ_2 / ps	c_2
$CsSnI_3$	96	2.3	1100	3.3	170	3.2	1900	1.6
$CsSn_{0.6}Ge_{0.4}I_3$	490	2.0	4200	1.7	410	1.1	3900	1.7

[a] *c*: Relative defect concentration, defined as the average number of defects per nanocrystal.

The fit from Poisson model returns the excited state lifetime of defect-free nanocrystal, though this is the least reliable result since the probability to find defect-free nanocrystals is low for both types of PNCs. In addition, the fact that the fit with two types of defects yields statistically good approximation (chi-square value close to 1) should not be interpreted as there are exactly two types of defects, but it is rather indication that there are different types of defects in each system. However, in order to compare the two systems, a sub-division of "fast" and "slow" quenching defects with respective fast and slow quenching components has been used. For the reference $CsSnI_3$ NCs, these time constants are around 96 and 1100 ps for the fast and slow components, respectively. With Poisson model fitting, we also determined the lifetimes of defect-free PNCs as 20 ± 8 ns and 43 ± 6 ns for $CsSnI_3$ NCs and $CsSn_{0.6}Ge_{0.4}I_3$ NCs, respectively. These results are plausible due to the low PLQY of the samples. According to the PL decay fit, the probability to find a defect-free nanocrystal is 0.4% for $CsSnI_3$ NCs and 2.5% for $CsSn_{0.6}Ge_{0.4}I_3$ NCs. These values are slightly higher compared to the measured PLQY, but the ratio between the two is identical.

2.7 Transient absorption (TA) spectra and decays of $CsSnI_3$ NCs and $CsSn_{0.6}Ge_{0.4}I_3$ NCs films

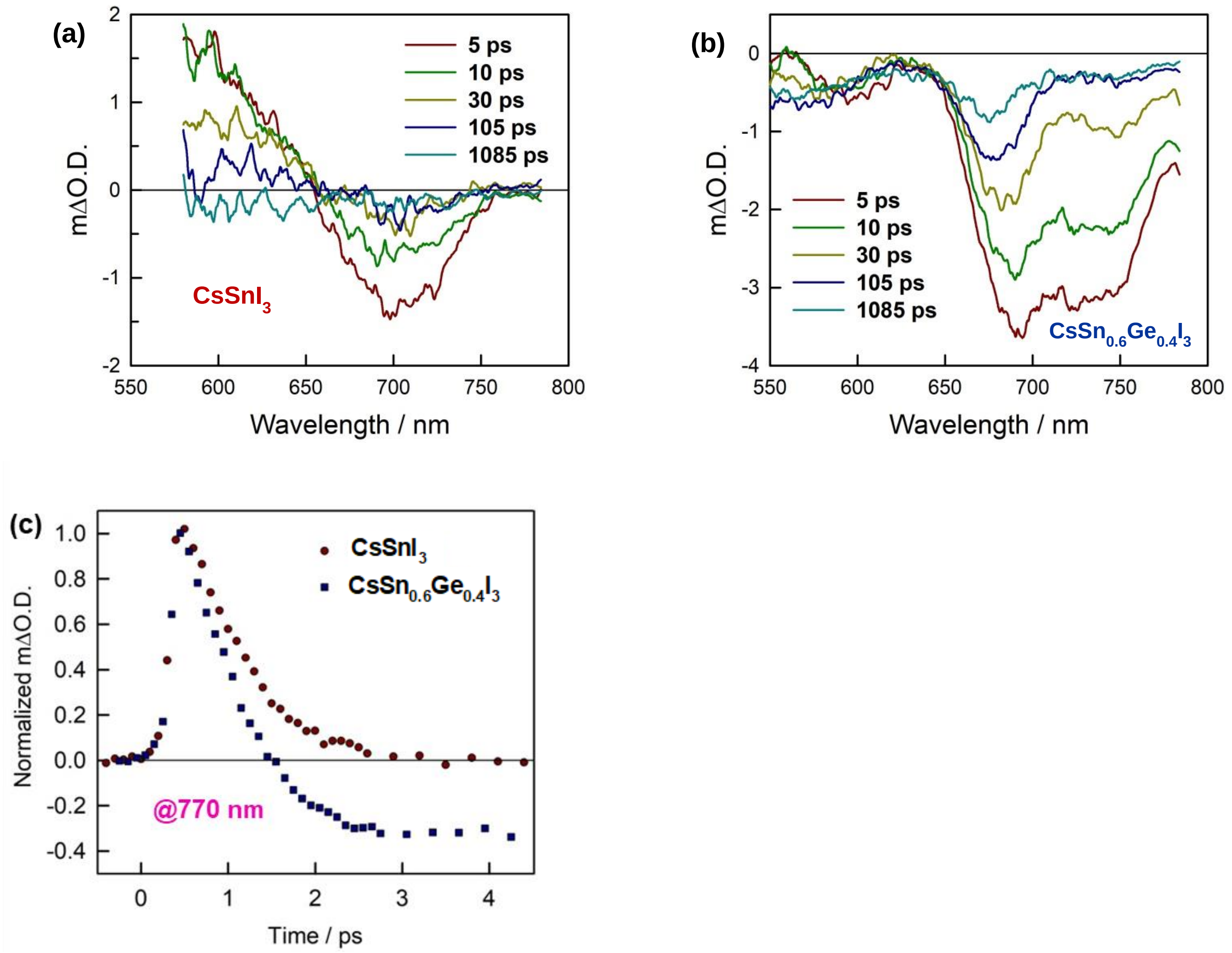

Figure S6. Ultrafast transient absorption (TA) spectrum of (a) CsSnI3 NCs and (b) $CsSn_{0.6}Ge_{0.4}I_3$ NCs in encapsulated film after variable time delays, excited at 500 nm with an excitation energy intensity of 10 μJ cm^{-2}. (c) TA decays of the encapsulated $CsSnI_3$ NCs and $CsSn_{0.6}Ge_{0.4}I_3$ NCs films, monitored at 770 nm.

2.8 Fitting results of the TA decays of $CsSnI_3$ NCs and $CsSn_{0.6}Ge_{0.4}I_3$ NCs films

Table S3. Time constants (τ_i) and contribution factor (A_i) for TA decays of the encapsulated $CsSnI_3$ NCs and $CsSn_{0.6}Ge_{0.4}I_3$ NCs films monitored at 700 nm, fitted with a bi-exponential function.

PNCs	τ_1 / ps	A_1 / %	τ_2 / ps	A_2 / %	τ_{avg}[a]
$CsSnI_3$	1.9	90.2	85.2	9.8	10.1
$CsSn_{0.6}Ge_{0.4}I_3$	18.0	89.5	2317.3	10.5	259.4

[a] $\tau_{avg} = (A_1\tau_1 + A_2\tau_2) / (A_1 + A_2)$

2.9 Morphology study of PNCs films

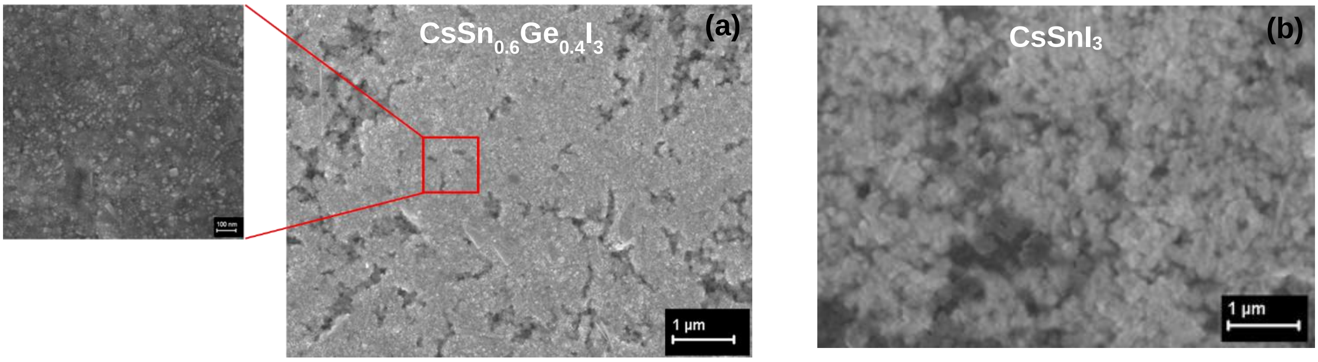

Figure S7. SEM images of (a) $CsSn_{0.6}Ge_{0.4}I_3$ NCs film and (b) $CsSnI_3$ NCs film observed at 3 kV, respectively. The inset in (a) shows a magnified image on selective surface area.

2.10 Optical stability of $CsSnI_3$ NCs and $CsSn_{0.6}Ge_{0.4}I_3$ NCs

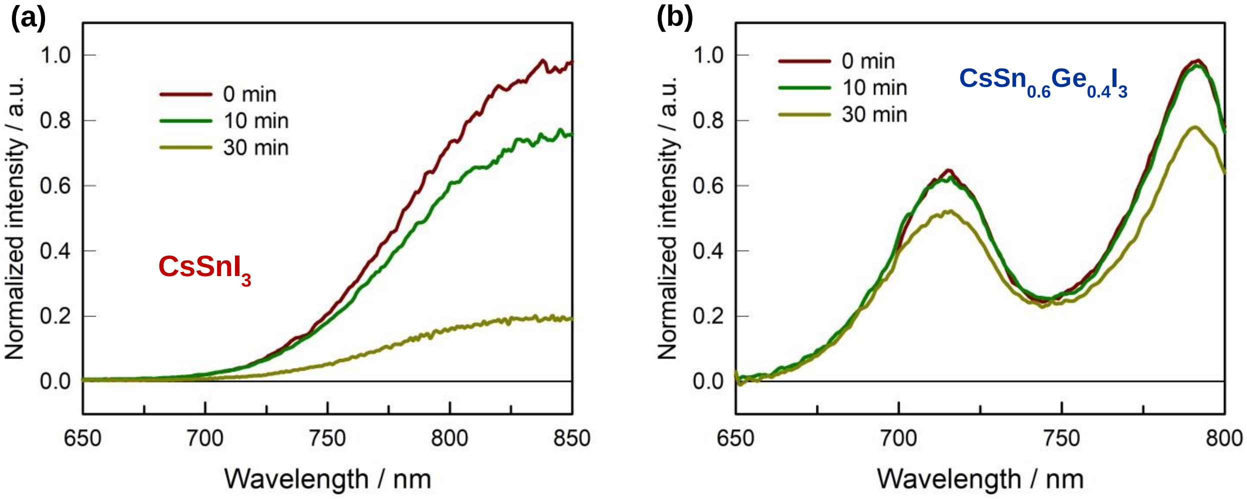

Figure S8. Time dependent PL spectrum of (a) $CsSnI_3$ NCs and (b) $CsSn_{0.6}Ge_{0.4}I_3$ NCs in suspension under ambient conditions.

2.11 Time-dependent XRD patterns of PNCs

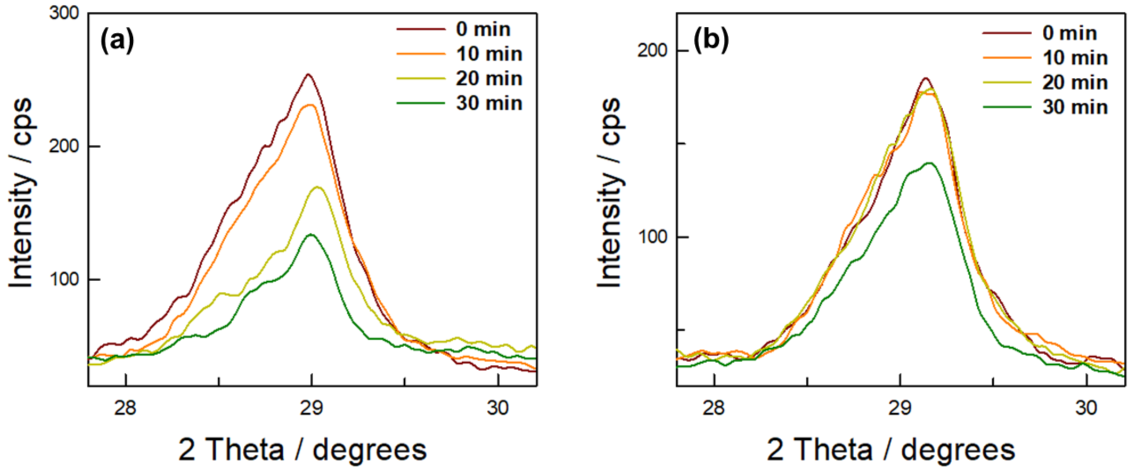

Figure S9. X-ray diffraction patterns of unencapsulated (a) $CsSnI_3$ NCs and (b) $CsSn_{0.6}Ge_{0.4}I_3$ NCs films before and after exposure for 10 min, 20 min and 30 min in ambient condition.

2.12 XPS analysis of aged $CsSn_{0.6}Ge_{0.4}I_3$ NCs

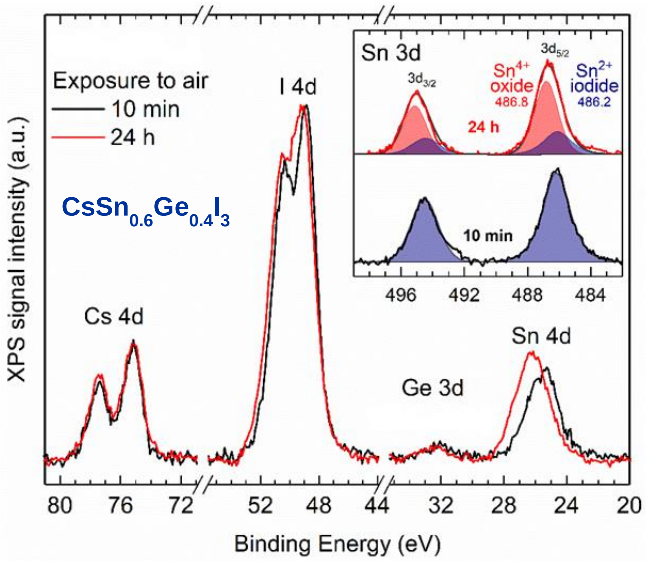

Figure S10. X-ray photoelectron spectroscopy (XPS) analysis of one $CsSn_{0.6}Ge_{0.4}I_3$ NCs film after 10 min and after 24 h air exposure. Insets depict elemental surface composition and component fitted Sn 3d transition.

References

[1] M. Liu, S. Makuta, S. Tsuda, S. Russo, S. Seki, J. Terao, Y. Tachibana, *J. Phys. Chem. C* **2017**, *121*, 25672-25681.

[2] H. Zhang, M. Liu, W. Yang, L. Judin, T. I. Hukka, A. Priimagi, Z. Deng, P. Vivo, *Adv. Mater. Interfaces* **2019**, *6*, 1–10.

Author Contributions

Conceptualization, M.L. (lead) and P.V. (lead); methodology, M.L. (lead) and P.V. (equal); data curation, M.L. (lead), H.P. (equal), H.A.-L. (equal), K.L. (equal), S.Q. (equal) and A.H. (equal); validation, M.L. (lead), H.A. (equal), K.L. (supporting), S.Q. (equal), A.H. (equal); formal analysis, M.L. (lead), P.V. (equal), H.P. (equal), H.A.-L. (equal), K.L. (supporting), S.Q. (equal), J.-H.S. (equal) and N.V.T. (equal); investigation, M.L. (lead), H.P. (equal), H.A.-L. (equal), K.L. (supporting), S.Q. (equal), A.H. (equal), J.-H.S. (supporting), N.V.T. (supporting) and P.V. (equal); funding acquisition, P.V. (lead), M.V. (equal), N.V.T. (supporting) and J.-H.S. (supporting); writing of original draft, M.L. (lead) and P.V. (equal); writing—review and editing, all authors (equal); project administration, P.V. (lead) and M.L. (equal).
